# Analysis of Aminoglycoside Modifying Enzyme Genes Responsible for High-Level Aminoglycoside Resistance among Enterococcal Isolates

**DOI:** 10.1155/2017/3256952

**Published:** 2017-12-24

**Authors:** Vishal Shete, Naveen Grover, Mahadevan Kumar

**Affiliations:** Department of Microbiology, Armed Forces Medical College, Pune, India

## Abstract

Enzymatic modification results in high-level resistance to aminoglycoside (HLAR), which eliminates the synergistic bactericidal effect of combined exposure to a cell wall-active agent and an aminoglycoside. So aim of the study was to determine prevalence of HLAR enterococcal isolate and to study distribution of aminoglycoside modifying enzyme genes in them. A total of 100 nonrepeat isolates of enterococci from various clinical samples were analyzed. As per Clinical and Laboratory Standards Institute guidelines enterococci were screened for HLAR by Kirby-Bauer disc diffusion method. Minimum inhibitory concentration of all isolates for gentamicin and streptomycin was determined by E-test. Multiplex polymerase chain reaction (PCR) was carried out for HLAR enterococcal isolates to identify aminoglycoside modifying enzymes genes responsible for resistance. 60% isolates were found to be high-level gentamicin resistant (HLGR) whereas 45% isolates were found to be high-level streptomycin resistant (HLSR). By multiplex PCR 80% HLGR isolates carried bifunctional aminoglycoside modifying enzyme gene* aac(6*′*)-Ie-aph(2*′′*)-Ia *whereas 18 out of 45 high-level streptomycin resistant, that is, 40%, isolates carried* aph(3*′*)-IIIa. *However,* aph(2*′′*)-Ib, aph(2*′′*)-Ic, aph(2*′′*)-Id, *and* ant(4*′*)-Ia *genes which encode other aminoglycosides modifying enzymes were not detected. Bifunctional aminoglycoside modifying enzyme gene* aac(6*′*)-Ie-aph(2*′′*)-Ia *is the predominant gene responsible for HLAR.

## 1. Introduction

Enterococci are natural inhabitants of the intestinal tract of many warm-blooded animals. As a result, they are released in large amounts with faeces and may become the predominant contaminant microbiota in many foods [1]. Enterococci are the third leading cause of infective endocarditis, accounting for 6-7% of prosthetic valve endocarditis and 5–20% of cases of native valve IE [2]. Nosocomial surveillance data from October 1986 to April 1997 list enterococci as the third leading cause of nosocomial bacteremia, accounting for 12.8% of all isolates [3]. A link between the use of antibiotics in animal husbandry and the rise of antibiotic resistance has been demonstrated. The resistance of enterococci in food animals is very similar to what has been described of enterococci isolated from nosocomial infections (including resistance to aminoglycosides, lincosamides, macrolides, nitrofurans, penicillins, quinolones, streptogramins, tetracycline, and rarely vancomycin) [4].

Currently, in* Enterococcus *genus there are 28 species [5]. Most of these species are not commonly found in humans.* Enterococcus faecalis *is the most common isolate, being associated with 80–90% of human enterococcal infections.* Enterococcus faecium *ranks second and is isolated from 10–15% of infections. Other enterococcal species, including* E. casseliflavus, E. avium, E. durans, E. cecorum, E. gallinarum, E. hirae, E. raffinosus, E. malodoratus, E. dispar, E. flavescens, *and* E. mundtii, *are infrequently isolated from human infections [6].

All enterococci have intrinsic low-level resistance to aminoglycosides, with minimal inhibitory concentrations (MICs) ranging from 4 *μ*g/mL to as high as 256 *μ*g/mL. The facultative anaerobic metabolism of enterococci is thought to produce their low-level resistance to all aminoglycosides by limiting drug uptake, which is associated with the proteins involved in electron transport. The addition of an agent that interferes with cell wall synthesis, such as ampicillin (or vancomycin), markedly increases uptake of the aminoglycoside, greatly enhancing the killing of the* Enterococcus* [3]. The aminoglycosides, gentamicin, and streptomycin are the only two compounds recommended for achieving this synergistic effect in clinical practice. The use of other aminoglycosides for this purpose is discouraged. High-level resistance (HLR) to aminoglycosides is defined by growth at concentrations of 2000 mg/L and 500 mg/L of streptomycin and gentamicin, respectively, on brain heart infusion (BHI) agar or 1000 mg/L of streptomycin when using BHI broth [7].

Enzymatic modification is the most common type of aminoglycoside resistance. Over 50 different enzymes have been identified. Enzymatic modification results in high-level resistance [8], which eliminates the synergistic bactericidal effect of combined exposure to a cell wall-active agent and an aminoglycoside [9]. It is hypothesized that the enzymes are derived from organisms that make the aminoglycoside or from the mutation of genes that encode the enzymes involved in cellular respiration [10]. There are three types of aminoglycoside modifying enzymes: (1) N-Acetyltransferases (AAC) which catalyze acetyl-CoA-dependent acetylation of an amino group; (2) O-Adenylyltransferases (ANT) which catalyze ATP-dependent adenylation of hydroxyl group; (3) O-Phosphotransferases (APH) which catalyze ATP-dependent phosphorylation of a hydroxyl group. Aim of the study was to determine prevalence of HLAR enterococcal isolate and to study distribution of aminoglycoside modifying enzyme genes in them.

## 2. Materials and Methods

The study was carried out in a tertiary care centre between January 2013 and January 2016. This study was carried out on 100 consecutive, nonrepeat isolates of enterococci isolated from various clinical samples received in the microbiology laboratory in a tertiary care centre. Specimens like pus, blood, urine, central line tip, and various others were collected aseptically and transported as per standard protocol.

### 2.1. Phenotypic Identification

**Figure 1:**
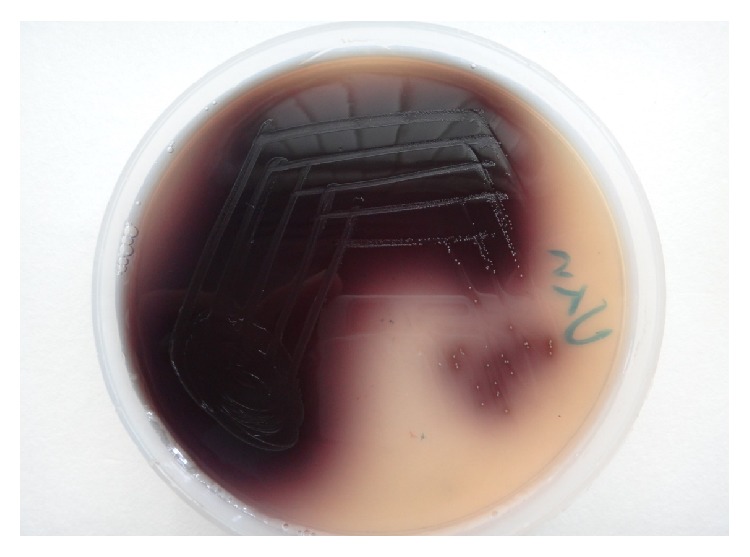
Bile Esculin Agar showing diffuse blackening of the medium by* Enterococcus* spp.

The isolates of enterococci were identified and speciated on the basis of colony morphology, Gram stain, and various biochemical reactions such as catalase test, bile esculin test (as shown in Figure 1), growth in 6.5% NaCl, PYR test, mannitol fermentation, arginine dihydrolase test, sucrose fermentation, arabinose fermentation, growth in pyruvate, lactose fermentation, and pigment production.

*Enterococcus faecium* and* Enterococcus faecalis *were further confirmed by PCR analysis using specific *ddl*_*E*.  *faecium*_ and *ddl*_*E*.  *faecalis*_ genes, respectively [11]. All enterococcal isolates were tested for their susceptibility to various antibiotics active against enterococci species by Kirby-Bauer method as per CLSI guideline 2013.

### 2.2. Testing for HLAR

**Figure 2:**
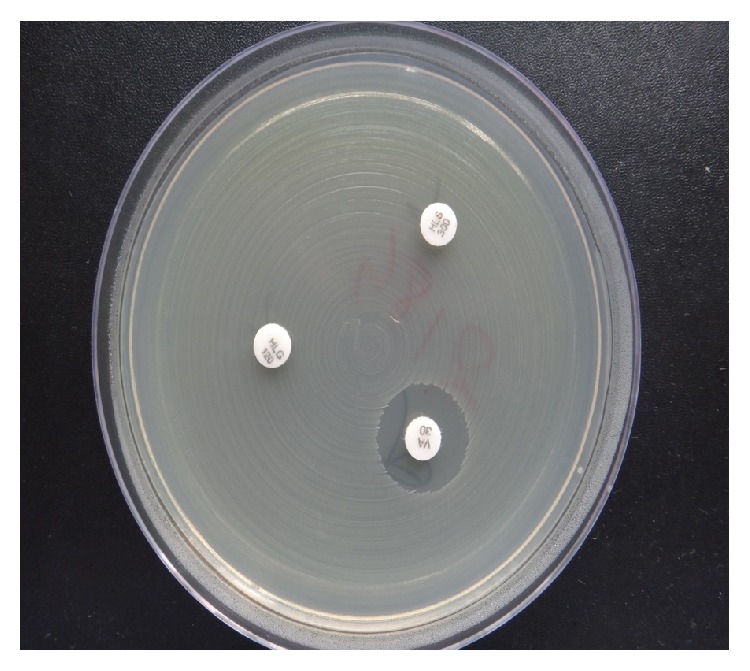
Testing of HLGR with HLG 120 *μ*g disc and HLSR with HLS 300 *μ*g disc.

Screening of HLGR with HLG 120 *μ*g disc and HLSR with HLS 300 *μ*g disc was done (as shown in Figure 2). Two National Committees for Clinical and Laboratory Standards Institute (CLSI) recommended QC strains,* E. faecalis *ATCC 29212 susceptible strain, and* E. faecalis *ATCC 51299 resistant strain.

**Figure 3:**
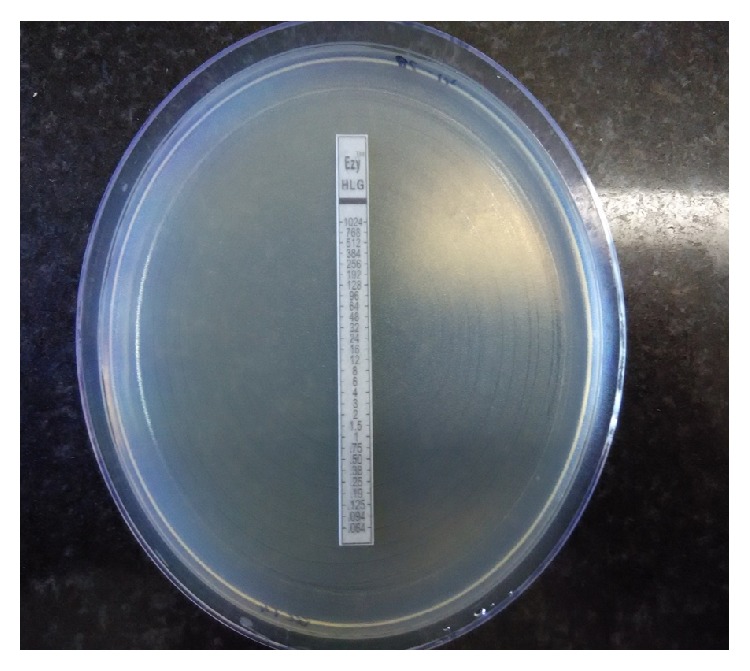
E-test showing MIC of gentamicin against resistant strain* Enterococcus faecium* (MIC > 1000 *μ*g/ml).

MIC of HLGR and HLSR enterococcal isolates was determined by E-test (Epsilometer Test; 0.064–1024 *μ*g/ml) (as shown in Figure 3).

### 2.3. Aminoglycoside Modifying Enzymes (AMEs) Genes Characterization

QIAamp DNA mini kits from QIAGEN, Germany, was used for DNA extraction. All HLAR isolates were subjected to multiplex PCR using 6 sets of primers. Oligonucleotide primers used are shown in Table 1.

The genes analyzed in the present study were* aac(6*′*)-Ie-aph(2*′′*)-Ia, aph(3*′*)-IIIa, aph(2*′′*)-Ib, aph(2*′′*)-Ic, aph(2*′′*)-Id, *and* ant(4*′*)-Ia*, responsible for high-level aminoglycoside resistance in enterococci.

PCR reactions were performed in a volume of 50 *μ*l with the following in a reaction tube: 5 *μ*l of DNA template, 1.5 mM MgCl2, 0.1 mM (each) deoxynucleoside triphosphate, 1x PCR buffer, and 2.5 U of* Taq *DNA polymerase, and the amount of each primer in the PCR was as follows: 25 pmol for* aac(6)-Ie-aph(2)-Ia*, 25 pmol for* aph(2)-Ib*, 3.5 pmol for* aph(2)-Ic*, 5 pmol for* aph(2)-Id*, 3 pmol for* aph(3)-IIIa*, and 2 pmol for* ant(4)-Ia *[9]. PCR was performed in a (Perkin-Elmer Gene Amp 2400) thermal cycler with an initial denaturation step of 3 min at 94°C; 35 cycles of 40 s at 94°C, 40 s at 55°C, and 40 s at 72°C; and a final extension step of 2 min at 72°C [9]. PCR products were analyzed by electrophoresis at 100 V for 1 to 1[1/2] hours on a 1% agarose gel stained with ethidium bromide.

Post-amplification analysis is done with gel electrophoresis with a 100-base pair molecular weight marker. The gel was viewed under UV transilluminator and was documented with the help of digital camera attached to the transilluminator and to the computer. After multiplex PCR, the amplicons were sent for confirmation by sequencing. The sequencing method employed was Sanger's capillary sequencing. The sequence was analyzed with the BLAST program from the National Center for Biotechnology Information (NCBI).

## 3. Results

Out of the 100 isolates, 52 (52%) were* E. faecalis *and 48 (48%) were* E. faecium *by conventional phenotypic and PCR analysis. The most common clinical sample from which enterococci were isolated was urine (60%) followed by blood (15%), pus (17%), tracheal aspirate (4%), semen (2%), and drain fluid (2%).

By Kirby-Bauer disc diffusion method 50% isolates showed resistance to ampicillin (10 *μ*g), 64% isolates showed resistance to ciprofloxacin (5 *μ*g), and 12% isolates showed resistance to vancomycin (30 *μ*g) and teicoplanin (30 *μ*g).

A total of 60 isolates were found to be high-level gentamicin resistant using 120 *μ*g disc by Kirby-Bauer disc diffusion method whereas 45 isolates were also found to be high-level resistance to streptomycin 300 *μ*g disc by Kirby-Bauer disc diffusion method as per CLSI guidelines. Thirty-seven (77%) out of 48 isolates of* E. faecium *and 23 (44%) out of 52 isolates of* E. faecalis *were found to be resistant to HLGR whereas 25 (52%) out of 48 isolates of* E. faecium *and 20 (38%) out of 52 isolates of* E. faecalis *were found to be resistant to HLSR.

All 60 isolates that were found to be high-level gentamicin resistant by Kirby-Bauer disc diffusion method were showing MIC > 500 *μ*g/ml by E-test. All 45 high-level streptomycin resistance isolates by Kirby-Bauer disc diffusion method were showing MIC > 1000 *μ*g/ml by E-test.

**Figure 4:**
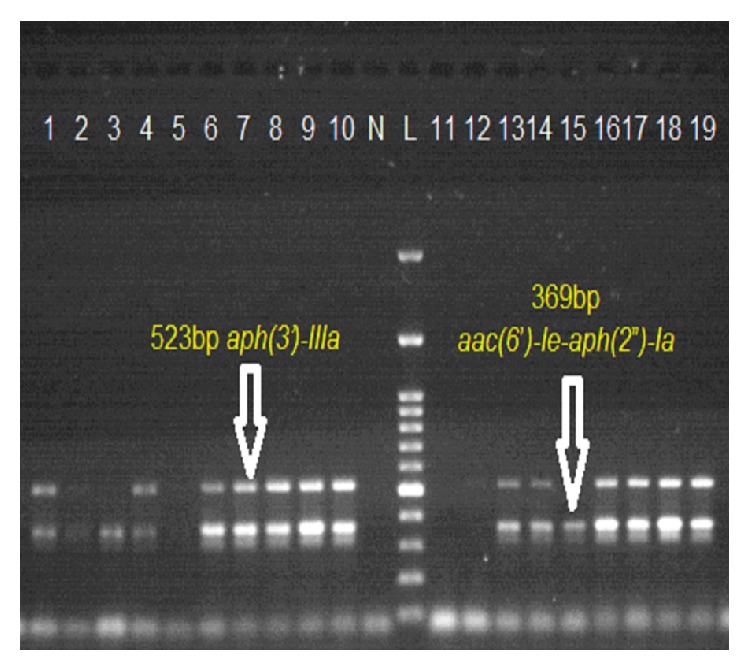
Representative image of gel electrophoresis of PCR for detecting Aminoglycoside modifying enzyme (AME) genes. Lane 1: positive control (*E. faecium* ATCC 51299). Lane N: negative control (*E. faecium* ATCC 29212). Lanes 4, 6, 7, 8, 9, 10, 13, 14, 16, 17, 18, and 19: positive; showing band at 523 bp* aph(3*′*)-IIIa* gene and 369 bp* aac(6*′*)-Ie-aph(2*′′*)-Ia gene*. Lane L: molecular marker (100 bp).

Forty-eight out of 60 HLGR, that is, 80%, isolates carried bifunctional AME gene* aac(6*′*)-Ie-aph(2*′′*)-Ia *whereas 18 out of 45 HLSR, that is, 40%, isolates carried* aph(3*′*)-IIIa *(as shown in Figure 4). However* aph(2*′′*)-Ib, aph(2*′′*)-Ic, aph(2*′′*)-Id, *and* ant(4*′*)-Ia *genes which encode other AMEs were not detected in our study. 30 HLGR* E. faecium *isolates carried* aac(6*′*)-Ie-aph(2*′′*)-Ia *gene and 18 HLGR* E. faecalis *carry bifunctional* aac(6*′*)-Ie-aph(2*′′*)-Ia *gene. Eleven HLSR* E. faecium *isolates carried* aph(3*′*)-IIIa *and 07 HLGR* E. faecalis *carried* aph(3*′*)-IIIa*.

Result of sequence blast on NCBI site showed 100% identity with bifunctional aminoglycoside modifying enzyme.

## 4. Discussion 

In the present study, the most common clinical sample from which enterococci were isolated was urine (60%) followed by pus (17%), blood (15%), tracheal aspirate (4%), semen (2%), and drain fluid (2%). Similar findings were also obtained in other studies, such as Mathur et al. [12] who obtained 49% of enterococci from urine samples.

In various studies,* E. faecalis *has been the predominant enterococcal species accounting for 80–85% of clinical isolates, followed by* E. faecium *which accounts for about 10–15% of clinical isolates [13]. But in recent years* E. faecium* has become more common, probably because of its greater antibiotic resistance. In the present study, out of 100 isolates, 52 (52%) were* E. faecalis *and 48 (48%) were* E. faecium *which is similar to other studies like Elango Padmasini et al. who obtained* E. faecalis *86/178 (48.3%) and* E. faecium* which was 80/178 (44.9%) [14].

Enterococci were traditionally regarded as low-grade pathogens but have emerged as an increasingly important cause of nosocomial infections in the 1990s. These infections are recognized by 3 t's: tough, tenacious, and troublesome [13, 15]. Furthermore, enterococci have assumed greater importance because of their increasing resistance to many antimicrobial agents, especially aminoglycoside, which include gentamicin and streptomycin.

Monotherapy for endocarditis with a *β*-lactam antibiotic (to which many enterococci are tolerant) has produced disappointing results, with low cure rates at the end of therapy. However, the addition of an aminoglycoside to a cell wall-active agent (a *β* lactam or a glycopeptide) increases cure rates and eradicates the organisms; moreover, this combination is synergistic and bactericidal in vitro. Therefore, combination therapy with a cell wall-active agent and an aminoglycoside is the standard of care for endovascular infections caused by enterococci [16]. This synergistic effect can be explained, at least in part, by the increased penetration of the aminoglycoside into the bacterial cell, presumably as a result of cell wall alterations attributable to the *β* lactam or glycopeptide. Nonetheless, attaining synergistic bactericidal activity in the treatment of severe enterococcal infections has become increasingly difficult because of the development of resistance to virtually all antibiotics available for this purpose [16].

Among the *β*-lactam agents, the most active are the aminopenicillin (ampicillin, amoxicillin) and ureidopenicillin (i.e., piperacillin); next most active are penicillin and imipenem. Against* E. faecium*, a combination of high-dose ampicillin (up to 30 g/d) and an aminoglycoside has been suggested even for ampicillin resistant strains if the MIC is <64 *μ*g/mL since a plasma ampicillin concentration of >100 *μ*g/mL can be achieved at high doses [16].

Out of 100 isolates, 60 (60%) showed high-level gentamicin resistance by disc diffusion method and by gentamicin E-test which is similar to other studies like Randhawa et al. who reported 68% HLGR [17]; a very recent study conducted in Iran [18] had reported around 60.45% HLGR strains in their region. Out of 100 isolates, only 45 (45%) showed high-level streptomycin resistance by disc diffusion method and by streptomycin E-test which is similar to other studies like Randhawa et al. [17]. who reported 43% HLSR. Apart from disc diffusion, CLSI recommends two more methods for HLAR detection, namely, agar dilution and broth microdilution.

It has been found in various studies that* E. faecium *accounts for far more resistance to high-level gentamicin and streptomycin. In present study HLGR in* E. faecium *is significantly higher (*P* value < 0.05) as compared to* E. faecalis *and HLSR in* E. faecium *is higher than* E. faecalis* but not statistically significant (*P* value > 0.05).

High-level aminoglycoside resistance among enterococci is due to the production of aminoglycoside modifying enzymes (AMEs) such as 2′-phosphotransferase, 3′-phosphotransferase, 6′-acetyltransferase, and 6′-adenyltransferase. In our study 48 out of 60 HLGR, that is, 80%, isolates carried bifunctional AME gene* aac(6*′*)-Ie-aph(2*′′*)-Ia *which is consistent with Padmasini et al. [14], and Hasani et al. [18], who reported 68.4% and 100% presence of bifunctional AME gene, respectively. Other studies also indicated that* aac(*6′*)-Ie-aph(2*′′*)-Ia *is the most prevalent gene among the gentamicin resistant enterococci [19, 20], whereas 18 out of 45 HLSR, that is, 40%, isolates carried* aph(3*′*)-IIIa. *Padmasini et al. [14] reported that 77% HLSR isolates carried* aph(3*′*)-IIIa* gene. However* aph(2*′′*)-Ib, aph(2*′′*)-Ic, aph(2*′′*)-Id, *and* ant(4*′*)-Ia *genes also found to encode high-level resistance to gentamicin (>500 g/mL) [14] were not detected in our study.

## 5. Conclusion

The prevalence of high-level gentamicin resistance among enterococci isolates in this study is 60% and prevalence of high-level streptomycin resistance was 45%.* E. faecium *and* E. faecalis *were almost equal in number but the resistance was found to be more common in* E. faecium* than in* E. faecalis*. Results obtained by E-test were similar to disc diffusion test. Multiplex PCR can detect AMEs genes with high sensitivity and specificity responsible for HLAR. Bifunctional AME gene* aac(6*′*)-Ie-aph(2*′′*)-Ia *is the predominant gene responsible for HLAR which eliminates the synergistic bactericidal effect of combined exposure to a cell wall-active agent and an aminoglycoside.

## Figures and Tables

**Table 1:** Primers used in the multiplex PCR [9].

Aminoglycoside resistance gene	Product size (bp)	Sequence type	Primer sequence (5′ → 3′)
*aac(6*′*)-Ie-aph(2*′′*)-Ia*	369	Fw	CAGGAATTTATCGAAAATGGTAGAAAAG
		R	CACAATCGACTAAAGAGTACCAATC
*aph(2*′′*)-Ib*	867	Fw	CTTGGACGCTGAGATATATGAGCAC
		R	GTTTGTAGCAATTCAGAAACACCCTT
*aph(2*′′*)-Ic*	444	Fw	CCACAATGATAATGACTCAGTTCCC
		R	CCACAGCTTCCGATAGCAAGAG
*aph(2*′′*)-Id*	641	Fw	GTGGTTTTTACAGGAATGCCATC
		R	CCCTCTTCATACCAATCCATATAACC
*aph(3*′*)-IIIa*	523	Fw	GGCTAAAATGAGAATATCACCGG
		R	CTTTAAAAAATCATACAGCTCGCG
*ant(4*′*)-Ia*	294	Fw	CAAACTGCTAAATCGGTAGAAGCC
		R	GGAAAGTTGACCAGACATTACGAACT

Fw: forward primer; R: reverse primer.
